# Automatic Extraction of Medication Mentions from Tweets—Overview of the BioCreative VII Shared Task 3 Competition

**DOI:** 10.1093/database/baac108

**Published:** 2023-02-03

**Authors:** Davy Weissenbacher, Karen O’Connor, Siddharth Rawal, Yu Zhang, Richard Tzong-Han Tsai, Timothy Miller, Dongfang Xu, Carol Anderson, Bo Liu, Qing Han, Jinfeng Zhang, Igor Kulev, Berkay Köprü, Raul Rodriguez-Esteban, Elif Ozkirimli, Ammer Ayach, Roland Roller, Stephen Piccolo, Peijin Han, V G Vinod Vydiswaran, Ramya Tekumalla, Juan M Banda, Parsa Bagherzadeh, Sabine Bergler, João F Silva, Tiago Almeida, Paloma Martinez, Renzo Rivera-Zavala, Chen-Kai Wang, Hong-Jie Dai, Luis Alberto Robles Hernandez, Graciela Gonzalez-Hernandez

**Affiliations:** Department of Computational Biomedicine, Cedars-Sinai Medical Center, Los Angeles, CA, USA; DBEI, The Perelman School of Medicine, University of Pennsylvania, Philadelphia, PA, USA; DBEI, The Perelman School of Medicine, University of Pennsylvania, Philadelphia, PA, USA; Department of Computer Science and Information Engineering, National Central University, No. 300, Zhongda Rd, Zhongli District, Taoyuan 320, Taiwan; Department of Computer Science and Information Engineering, National Central University, No. 300, Zhongda Rd, Zhongli District, Taoyuan 320, Taiwan; IoX Center, National Taiwan University, Da’an District, Section 4, Roosevelt Rd, No. 1, Barry Lam Hall, Taipei 106, Taiwan; Research Center for Humanities and Social Sciences, Academia Sinica, No. 128, Section 2, Academia Rd, Nangang District, Taipei 115, Taiwan; Computational Health Informatics Program, Boston Children’s Hospital, Boston, MA, USA; Department of Pediatrics, Harvard Medical School, Boston, MA, USA; Computational Health Informatics Program, Boston Children’s Hospital, Boston, MA, USA; Department of Pediatrics, Harvard Medical School, Boston, MA, USA; NVIDIA, Santa Clara, CA, USA; NVIDIA, Santa Clara, CA, USA; Department of Statistics, Florida State University, Tallahassee, FL, USA; Department of Statistics, Florida State University, Tallahassee, FL, USA; Data and Analytics Chapter, F. Hoffmann-La Roche Ltd, Switzerland; Data and Analytics Chapter, F. Hoffmann-La Roche Ltd, Switzerland; Pharmaceutical Research and Early Development, Roche Innovation Center Basel, Switzerland; Data and Analytics Chapter, F. Hoffmann-La Roche Ltd, Switzerland; Speech and Language Technology Lab, DFKI, Berlin, Germany; Speech and Language Technology Lab, DFKI, Berlin, Germany; Department of Biology, Brigham Young University, Provo, UT, USA; Department of Computational Medicine and Bioinformatics, Medical School, University of Michigan, Ann Arbor, MI, USA; Department of Learning Health Sciences, Medical School, University of Michigan, Ann Arbor, MI, USA; School of Information, University of Michigan, Ann Arbor, MI, USA; Department of Computer Science, Georgia State University, Atlanta, GA, USA; Department of Computer Science, Georgia State University, Atlanta, GA, USA; CLaC Labs, Concordia University, Montreal, Canada; CLaC Labs, Concordia University, Montreal, Canada; DETI, Institute of Electronics and Informatics Engineering of Aveiro, University of Aveiro, Portugal; DETI, Institute of Electronics and Informatics Engineering of Aveiro, University of Aveiro, Portugal; Department of Computation, University of A Coruña, Spain; Computer Science and Engineering Department, Universidad Carlos III de Madrid, Madrid, Spain; Computer Science and Engineering Department, Universidad Carlos III de Madrid, Madrid, Spain; Big Data Laboratory, Chunghwa Telecom Laboratories, Taoyuan, Taiwan; Department of Computer Science, National Yang Ming Chiao Tung University, Hsinchu, Taiwan; Department of Electrical Engineering, College of Electrical Engineering and Computer Science, National Kaohsiung University of Science and Technology, Kaohsiung, Taiwan; Department of Computer Science, Georgia State University, Atlanta, GA, USA; Department of Computational Biomedicine, Cedars-Sinai Medical Center, Los Angeles, CA, USA

## Abstract

This study presents the outcomes of the shared task competition BioCreative VII (Task 3) focusing on the extraction of medication names from a Twitter user’s publicly available tweets (the user’s ‘timeline’). In general, detecting health-related tweets is notoriously challenging for natural language processing tools. The main challenge, aside from the informality of the language used, is that people tweet about any and all topics, and most of their tweets are not related to health. Thus, finding those tweets in a user’s timeline that mention specific health-related concepts such as medications requires addressing extreme imbalance. Task 3 called for detecting tweets in a user’s timeline that mentions a medication name and, for each detected mention, extracting its span. The organizers made available a corpus consisting of 182 049 tweets publicly posted by 212 Twitter users with all medication mentions manually annotated. The corpus exhibits the natural distribution of positive tweets, with only 442 tweets (0.2%) mentioning a medication. This task was an opportunity for participants to evaluate methods that are robust to class imbalance beyond the simple lexical match. A total of 65 teams registered, and 16 teams submitted a system run. This study summarizes the corpus created by the organizers and the approaches taken by the participating teams for this challenge. The corpus is freely available at https://biocreative.bioinformatics.udel.edu/tasks/biocreative-vii/track-3/. The methods and the results of the competing systems are analyzed with a focus on the approaches taken for learning from class-imbalanced data.

## Motivation

Twitter posts are now recognized as an important source of patient-generated data, providing unique insights into population health. A fundamental step toward incorporating Twitter data in pharmacoepidemiological research is to automatically recognize medication mentions in tweets. This is often the first step of complex natural language processing pipelines. A common approach is to search for tweets containing lexical matches of medication names occurring in existing lexicons such as RxNorm (https://www.nlm.nih.gov/research/umls/rxnorm/docs/rxnormfiles.html, accessed 27 October 2022) and the Food and Drug Administration Orange Book (https://www.fda.gov/drugs/drug-approvals-and-databases/approved-drug-products-therapeutic-equivalence-evaluations-orange-book, accessed 27 October 2022). This approach has several limitations, even when allowing for variants and misspellings. In a prior study, (1), when using the lexical match approach on a corpus where names of medications are rare, the authors retrieved only 71% of the tweets manually identified as mentioning a medication, and more than 45% of the tweets retrieved were false positives. For example, when tweets mention the word ‘propel’, it denotes predominantly the verb and not the brand name of a corticosteroid. In addition, descriptive text and types of medications (such as ‘my blood pressure med’ or ‘my anti-seizure pill’), as well as references to compounds and ‘street’ names for medications (such as ‘the blue pill’), are not present even in extensive lexicons and may be important to derive potential diagnosed conditions. The BioCreative VII Task 3 shared task presented an opportunity to go beyond the lexical match approach, fostering the development of new methods for the extraction of medications mentioned in posts and enhancing the utility of social media for public health research.

Existing work tackling the problem of detecting medication names on Twitter has focused on collecting large corpora to train machine learning systems (and not mentions of medications in a user’s timeline). In (2–4), the authors collected tweets mentioning a medication in a predefined list. This results in selection bias: ‘you only collect what is on the list’. Other studies that used the keyword-based collection removed all tweets mentioning common phrases that could be confused with medication names (5) or imposed that a medication name co-occurs with the name of a disease (6) in an attempt to reduce the ‘noise’ in their collections. By discarding ambiguous tweets, these approaches miss valuable examples for training machine learning algorithms. Ambiguous tweets are very frequent and are often mislabeled by automatic systems, as systems not exposed to them would tend to assign too much weight to the features representing the medication names and too little to the features representing the linguistic context around the medication names. In short, any approach that tries to reduce ‘noise’ by making the problematic tweets ‘disappear’ from the corpus would have limited applications and would not be useful to train a generic system to detect medication mentions.

To reduce this selection bias during the competition, the organizers chose a corpus composed of 212 Twitter user timelines (181 607 tweets). This corpus was collected for an unrelated study (7) that imposed health-related criteria and collected the timelines of the users that met the criteria. The authors collected the corpus by first identifying users self-reporting a pregnancy; then, for those classified as true pregnancy announcements, they collected all their publicly available tweets using the Twitter application programming interface (API). They annotated all mentions of medications in the timelines. Using this corpus for the shared task ensures that it is representative of the way Twitter users mention their medications on the platform and exhibits a natural distribution of the tweets of interest. Hereafter, we refer to this corpus as the *SMM4HDrug corpus*, where SMM4H stands for Social Media Mining for Health. A limitation of this collection method is that the distribution of the tweets of interest and the other tweets is extremely imbalanced, with only 0.2% of the tweets in the SMM4HDrug corpus mentioning a medication. Such class imbalances are known to degrade the performance of machine learning systems when the training process is not modified to account for the imbalance (8, 9). Consequently, the class imbalance of the SMM4HDrug corpus was the main concern for the participants in the competition. They proposed concrete solutions to train their systems on this challenging dataset, developing systems capable of closely modeling the detection of medication names in tweets as one would expect to have them when complete timelines are analyzed.

In sections Task description and corpora and Evaluation, we describe the details of the BioCreative VII Task 3 competition and the evaluation method, respectively. In the section Systems, we summarize the results of the competition and review all competing systems. We discuss the strategies followed in the section Discussion and analyze what we believe to be the main features in the strategy to successfully solve the task. By comparing the 16 systems competing in the task, this study provides an up-to-date review of some of the techniques to extract information from social media.

## Task description and corpora

Task 3 was a named entity recognition (NER) task. The goal of the task was to detect tweets mentioning medication names (prescriptions and over-the-counter), or dietary supplements, and to extract the spans of text denoting them.

During the competition, the SMM4HDrug corpus was released. This corpus consists of 212 Twitter users’ timelines from the 44 825 timelines collected for a project described in (7), fully annotated with identified medication mentions. To detect the users, the Twitter streaming API was queried for keywords related to 14 patterns such as ‘I.*(m|a.m.|’m).*(weeks|months).*(pregnant)’ or ‘baby.*arriving’. A supervised classifier was trained to improve the precision of the patterns (for the training and evaluation details of the classifier see (10)). All publicly available tweets for the users identified were collected, both before and after the announcement of the pregnancy. For the competition, 212 users were randomly selected from this cohort and the spans of medications mentioned in all tweets posted during the pregnancy time frame were annotated. The pregnancy time frame is the time period starting 1 month before the pregnancy and extending to 1 month after the delivery. A senior annotator (KO) and a staff annotator double-annotated 12 timelines from the 212 timelines to compute the Inter-Annotator Agreement, which was strong, with 0.88 Cohen’s Kappa. This gold standard, the SMM4HDrug corpus, has a natural distribution of medication mentions on Twitter timelines, which is extremely imbalanced, with only 442 tweets out of 181 607 mentioning a medication or nutritional supplement. That is, only approximately 0.2% of the tweets are positive examples.

Table 1 shows some examples of the tweets annotated in the corpus. Each tweet is represented by its unique tweet ID, the text of the tweet and, when the tweet includes the mention of a medication or nutritional supplement, the starting and ending position of the mention in the text followed by the mention itself and the normalized name of the said medication or supplement. These values are left empty for the tweets not mentioning medications. When a tweet mentions multiple medication names, such as tweet 4 in the list of examples, the tweet is duplicated with each occurrence containing the span of one medication name.

**Table 1.** Examples of tweets annotated with medication mentions

Tweet ID	Text	Begin	End	Span	Medication normalized
1	Only 3 Arnica Balms left…	8	19	Arnica Balms	arnica balm
2	@user sudafed that I’m not sure I’m comfortable taking it	7	13	Sudafed	Sudafed
3	I like this song!	–	–	–	–
4	@user no my body hurts, they prescribed me hydros and moltrin	44	49	Hydros	Hydrocodone
4	@user no my body hurts, they prescribed me hydros and moltrin	55	61	Moltrin	Motrin

For Task 3, the organizers split the SMM4HDrug corpus into three sets, a training set (218 positive and 88 770 negative tweets), a validation set (93 positive and 38 044 negative tweets) and a test set (131 positive and 54 351 negative tweets). The organizers split the corpus by randomly selecting the tweets from all timelines so that the training, validation and test sets are likely to have tweets from all users’ timelines. Thus, there is no notion of a ‘timeline’ in the SMM4HDrug corpus, but the ratio of positives to negatives that occurred in actual timelines is respected.

In addition to the training and validation sets from the SMM4HDrug corpus, the organizers provided the participants with an extra set of 9622 tweets annotated with medication names, 4975 positive and 4647 negative tweets, hereafter called the SMM4HBalancedDrug corpus. The SMM4HBalancedDrug corpus is smaller than the training set but it is more balanced. They provided the SMM4HBalancedDrug corpus to the participants to help them train their machine learning systems with supervision. This corpus was first released in 2018 during the Social Media Mining for Health (#SMM4H’18) shared tasks (11) (https://healthlanguageprocessing.org/smm4h-2022/, accessed 27 October 2022). To collect the tweets of the SMM4HBalancedDrug corpus, four weak classifiers were developed and their predictions used to select tweets likely to mention a medication in the initial collection of the 44 825 users’ timelines. Overall, 9622 tweets were randomly selected for manual annotation. The Inter-Annotator Agreement was strong with a score of 0.892 Cohen’s Kappa, see (1) for details.

In March 2021, the organizers released to the participants the training and validation sets of the SMM4HDrug corpus as well as the SMM4HBalancedDrug corpus for the development of their systems. On 15 September 2021, they released the test set to the participants, who had 4 days to automatically predict the spans of medications in the test set. The participants submitted their predictions online to the competition site hosted in CodaLab (https://competitions.codalab.org/competitions/23 925). The competition has an evaluation script automatically triggered upon submission of a system’s predictions. The organizers allowed each team of participants to submit the predictions of a maximum of three different systems.

CodaLab Competitions is a web-based computing environment that allows researchers to share and interact with code, data and experiments in the context of online competitions. CodaLab is free and open-source, with a public instance of the server running at https://codalab.lisn.upsaclay.fr/ (accessed 27 October 2022). A CodaLab competition consists of the description of the competition, validation and test datasets, an evaluation script and an interactive interface that allow participants of the competition to upload their predictions of what the labels of the validation and test datasets should be. Upon reception of the predictions, CodaLab runs the evaluation script and computes the performances of the participants who have the choice to display their results on a public leaderboard or keep them private. Aside from not having to manually manage the participants’ submissions and their evaluation, since CodaLab runs these steps fully automatically, the main benefit of using CodaLab for Task 3 is that the competition could remain active even after the end of the BioCreative VII shared tasks event. Since the organizers continue to release the training and the unlabeled test datasets to any researcher making a request, CodaLab allows them to register, evaluate their systems and compare their results with others during the post-evaluation period, which is kept open indefinitely. This ensures a fair comparison when evaluating new approaches.

## Evaluation

### Metrics

The competing systems were evaluated using precision, recall and F1-score metrics for the positive class (i.e. the annotated spans of medication names). A true positive (TP) is a medication mention for which a system correctly predicted its span. That is an exact match on the starting and ending positions as compared to the gold standard. A false positive (FP) is a predicted medication mention that is not present in the gold standard. A false negative (FN) is a medication mention present in the gold standard but missed by the system. The precision *P*, given in equation (1), is the ratio of correct mentions (TP) to all mentions predicted by the system (TP + FP). The recall *R* (equation (2)) is the ratio of medication names successfully predicted out of all of the mentions in the gold standard that should have been detected. The F1-score $F1$ (equation (3)) is the harmonic mean of the precision and recall and summarizes the overall performance of a system.

(1) $$ P=TP/TP+FP $$

(2) $$ R=TP/TP+FN $$

(3) $$ F1=2*(P*R)/(P+R) $$

During the competition, strict and overlapping evaluations were performed. In the strict evaluation, a system was only rewarded if it predicted the exact beginning and end positions of the spans of the medication names in the gold standard. In the overlapping evaluation, this constraint was relaxed and the system was rewarded when it predicted a span that overlapped with a span of a medication name in the gold standard. For example, given Tweet 1 in Table 1, a system that predicts ‘Arnica’ (8–13) as a medication name is penalized with an FP under strict evaluation since the gold standard does not have ‘Arnica’ and with an FN since it missed ‘Arnica Balms’, which is in the gold standard. With overlapping evaluations, the system is rewarded with a TP prediction since ‘Arnica’ (8–13) is a substring of ‘Arnica Balms’ (8–19). The competition rules ranked the systems according to their strict F1-scores. When two systems achieved equal strict F1-scores, their overlapping F1-scores were used to decide the rank.

### Baseline System

At the beginning of the competition, code, documentation and trained models of a baseline extractor were released by the organizers to help participants start their development. Its detailed description and evaluation can be found in (12); we summarize the details of the system in this section.

The baseline system has two modules: a classifier and an extractor. The modules are applied sequentially. The classifier uses a disambiguated lexical match approach. Initially, tweets that match an entry of the lexicon are labeled 1 and 0 otherwise. The lexicon contains 44 498 medication names from RxNorm (13). Automatically generated variants of the medication names were added to the lexicon to account for misspellings using the method described in (1). The variants were manually curated to remove those that could be confused with common English words (such as ‘some’, a variant generated for ‘Sone’, a corticosteroid). The lexicon was also extended with 231 generic references to medication classes, such as ‘pain meds’, ‘statin’ or ‘antibiotic’. The generic references were manually compiled. In (12), the authors found that the lexical match approach alone for classification achieved good recall (0.756) but low precision (0.253) due to the ambiguity of some entries in the lexicon. Thus, they improved the performance of the classifier by training a (BERT) Bidirectional Encoder Representations from Transformers model to disambiguate the tweets selected by the lexicon match step. The BERT model was trained on SMM4HBalancedDrug to learn the linguistic contexts where ambiguous names are more likely to appear. The authors reported a performance of 0.795 F1-score (precision 0.875 and recall 0.728) for the classifier module when the BERT model disambiguated the lexicon entries detected. The second module performs the extraction of the medication names in the tweets predicted by the classifier as mentioning at least one medication or supplement name. The extractor was designed with a standard architecture for extraction: a BERT embedding layer followed by a bidirectional long short-term memory (BiLSTM) layer that predicts for each token of a tweet whether the token is inside or outside a medication mention. The extractor works downstream of the classifier and expects as inputs tweets with medication or supplement mentions. It was first pre-trained on the SMM4HBalancedDrug corpus to provide the system with examples of the patterns used when medications or supplements are mentioned and of ambiguous phrases with medication and supplement names. It was then fine-tuned on the tweets of the SMM4HDrug corpus training set filtered by the classifier. The performance of the baseline extractor is reported in Table 2. One may think that the classifier module is not needed since the extractor performs at the same time the detection of the tweets mentioning medication names and the extraction of their positions. However, empirical results in (1) indicate that separating the classification and the extraction steps may facilitate optimizing the system as a whole by optimizing each individually. The loss function of the classifier focuses on the semantics of health-related tweets and that of the extractor on detecting the spans of the medications.

**Table 2.** Strict/Overlapping F1-scores(F1), precision (P), recall (R) and system summaries

	Strict			Overlapping			
Team	F1	P	R	F1	P	R	System summary
1	**0.804**	0.799	0.810	**0.838**	0.832	0.844	Classifier + question answering; Classifier: ensemble BERTweet-large, data augmentation with MultNLI, TwiMed, SMM4H corpora, 2 million silver-standard tweets
2	**0.804**	0.799	0.810	0.824	0.819	0.830	Ensemble of BERT-based multi-task classifiers/extractors; data augmentation & generation with SMM4H’18 and 1194 silver-standard positive tweets
3	0.764	0.805	0.728	0.793	0.835	0.755	Ensemble of Megatron-BERT-345M extractors trained with out-of-fold
4	0.762	0.714	**0.816**	0.794	0.744	**0.850**	PubMedBERT-based extractor; data augmentation and generation with SMM4H’18 and 18 800 silver-standard tweets
Baseline	0.758	0.890	0.660	0.773	0.908	0.673	
5	0.738	0.850	0.653	0.762	0.876	0.673	BERT-base + fasttext embeddings + BiLSTM + CRF extractor; data augmentation with SMM4H’18
6	0.725	0.752	0.701	0.804	0.827	0.782	Ensemble of BERTweet and Twitter-RoBERTa extractors trained with out-of-fold; oversampling and data generation with SMM4H’18
7	0.725	0.786	0.673	0.777	0.841	0.721	BioRedditBERT extractor with post-filtering using a lexicon; undersampling and data augmentation with SMM4H’18
8	0.705	0.748	0.667	0.755	0.802	0.714	Extracter based on manually curated lexicons
9	0.689	0.678	0.701	0.775	0.755	0.796	DistilBERT extractor trained with bootstrapping; oversampling with SMM4H’18
10	0.687	0.771	0.619	0.737	0.831	0.662	Classifier + Lexicon; Classifier: BERT-large; data augmentation with 200 000 silver-standard tweets from SMM4H’17
11	0.683	0.629	0.748	0.739	0.680	0.810	Collaborative recurrent modules extractor, modules encode various features such as word clinicalBERT embedding, lexicon, POS and morphology
12	0.681	0.738	0.633	0.747	0.810	0.694	Twitter-RoBERTa + FCNs + CRF extractor with a weighted loss function
13	0.631	**0.910**	0.483	0.640	**0.923**	0.490	BERTweet-based extractor; data augmentation with 160 000 positive tweets from SMM4H’18, TwiMed, CADEC and silver-standard tweets
14	0.606	0.731	0.517	0.704	0.840	0.605	BERT-based extractor; data augmentation with SMM4H’18 and 10 500 silver-standard tweets
15	0.585	0.727	0.490	0.659	0.812	0.554	Classifier + extractor; Classifier: ensemble of BERT-based; Extractor: BERT-based; data augmentation with SMM4H’18 and 326 000 silver-standard tweets from past projects
16	0.548	0.634	0.483	0.638	0.741	0.561	Not available

## Systems

### Results

Sixty-five teams registered to participate in the shared task and sixteen submitted at least one prediction file on CodaLab. The organizers kept the best predictions for each submitting team. Table 2 presents the performance of each team and summarizes the architectures of the systems, the type of embeddings when available, as well as the strategies applied to train the systems to account for the extreme class imbalance. Four systems achieved better performance than the baseline system.

### Individual system descriptions

All participants who submitted their predictions during the evaluation period of the competition were invited to submit a technical summary of the strategy used for their participation in the BioCreative Task 3. Summaries of the 14 teams who submitted their predictions are presented below (We excluded Teams 5 and 16 from Table 3 since they did not submit a summary of their systems). They are listed by their respective performance in the shared task.

**Table 3.** Team numbers and system description papers

Team	Institution	Country	System description paper
1	National Central University/Academia Sinica	Taiwan	Zhang Y. *et al.* (14)
2	Boston Children’s Hospital/Harvard Medical School	USA	Xu D. *et al.* (15)
3	NVIDIA	USA	Anderson C. *et al.* (16)
4	Florida State University	USA	Han Q. *et al.* (17)
5			NA
6	Roche	Switzerland	Kulev I. *et al.* (18)
7	DFKI/Technische Universität Berlin/Université Paris-Saclay	Germany/France	Roller R. *et al.* (19)
8	Brigham Young University	USA	Piccolo S. (20)
9	University of Michigan	USA	Han P. *et al.* (21)
10	Georgia State University	USA	Tekumalla R. and Banda J. (22)
11	Concordia University	Canada	Bagherzadeh P. and Bergler S. (23)
12	University of Aveiro/University of A Coruña	Portugal/Spain	Silva J. *et al.* (24)
13	Universidad Carlos III de Madrid	Spain	Zavala R. *et al.* (25)
14	Chunghwa Telecom Laboratories	Taiwan	Lee Y-Q. *et al.* (26)
15	Georgia State University	USA	Hernandez L. *et al.* (27)
16			NA

#### Team 1: National Central University/Academia Sinica

For our participation in BioCreative VII Task 3, we developed a two-stage system consisting of two deep learning models: (i) the classification of whether a tweet contains medication names and (ii) the medication mention extraction.

For detecting whether a tweet contains one or more medication mentions, we did not treat it as a one-sentence text classification task; instead, we transformed it into a two-sentence textual entailment task by adding a prompting sentence ‘This tweet mention a medication, medication or dietary supplement in it’ in front of each tweet. The prompting sentence was manually crafted based on the task description. We chose BERTweet-large (28) pre-trained language model and used [CLS] token embedding as features to output the logits of positive and negative for the entailment task.

For medication mention extraction, we converted it from a sequential labeling task to an extractive question-answering (QA) task (29) by adding the query ‘Extract the spans that mention a medication, medication or dietary supplement in the tweet’. The query has medical keywords; therefore, transformers could utilize them to weight the tokens of a tweet in the self-attention mechanism. In addition, Splinter (30) was used as the model to extract the spans of medication names. Splinter’s architecture is based on BERT, which uses multiple layers of transformer encoder component; it uses a pre-training method, called recurring span selection, specifically designed for the extractive QA task.

To address the class imbalance and annotation inconsistency in the training dataset, we used a data-centric approach, including data augmentation with external datasets and annotation correction. The tweets containing medications in the four external datasets (TwiMed Dataset (4), SMM4H 2018—Task 2 Medication Intake Classification Dataset (11), SMM4H 2017—Task 1 ADR Classification Dataset (31) and SMM4H 2018—Task 1 Dataset (11)) were added to the training data. We used the Levenshtein distance algorithm and manual corrections to tackle the entity mismatching problem and task definition inconsistency problem of some external datasets.

During the evaluation phase, we selected seven BERTweet-large classification models with F1 scores higher than 0.9 on the validation set and used a voting-based ensemble method. For the extractive QA component, we only used the Splinter model, which had the highest performance. On the test set, our system achieved a strict F1-score of 0.804, which is above the average performance 0.696 of 16 participating teams.

### Team 2: Boston Children’s Hospital, Harvard Medical School

We approached the medication detection in tweets with a multi-tasks setting, where we jointly trained a binary classifier and a sequence labeling classifier with a shared transformer network. Our binary classifier takes the representation of the [CLS] token as input to classify whether a post contains a medication mention. Our sequence labeling classifier takes the representations of all sub-word tokens and outputs one Beginning, Inside, Outside (BIO) tag for each token. The final loss of our model is the sum of losses from the two classifiers. In contrast to the model just trained on the sequence labeling task, we showed that the jointly trained model can better leverage the context information for the sequence labeling classifier.

To mitigate the challenges of imbalanced training examples with no predefined medications in the shared task datasets (2021 data), we considered two ways to augment the data: (i) processing the existing dataset from the SMM4H-2018 shared task (11) (2018 data) to find the spans of the medication mentions from tweets and (ii) using the Twitter API to extract additional tweets (extra data) by keyword searching and replacing the medication keywords with newly collected medication mentions having similar medication use from a medication review platform, WebMD (https://www.webmd.com/drugs/2/index), to increase the coverage of different medication mentions.

In an attempt to improve performance over individual models, we applied an ensemble technique to combine the results of different models. We selected eight transformer models pre-trained with different settings as our initialization points: BERT-base-cased (32), BERT-base-uncased (32), BERT-large-cased (32), BERT-large-uncased (32), BioBERT-base-cased (33), BioRedditBERT-base-uncased (34), BioClinicalBERT-base-cased (35) and PubMedBERT-base-uncased (36). After fine-tuning the models on tweets, we aggregated the outputs from the above eight models (i.e. the spans of medication mentions) and selected predictions that have been agreed upon by multiple models, where the exact number is a parameter we tuned on the validation set.

Our best performance on the test set was achieved with an ensemble of models fine-tuned on 2021+2018+extra and agreed on by more than five models. This ensemble achieved the best strict F1 of 0.804 among all submissions, 4.6% points higher than the baseline system and 10.8% points higher than the mean of all participants (15).

### Team 3: NVIDIA

We fine-tuned multiple BERT-style (32) language models to perform token-level classification, using three token labels: B-DRUG, I-DRUG and O. These models included Megatron-BERT-345M (37) (a 345-million parameter model trained on general domain text), BioMegatron-BERT-345M (38) (trained on text from PubMed), RoBERTa-large (39) (trained on general domain text) and BERTweet-large (28) (trained on tweets). Of these models, we found that Megatron-BERT-345M with an uncased vocabulary gave the highest F1-scores when trained on the training set and evaluated on the validation set.

For our submissions, we created two different ensembles. The first ensemble consisted of five Megatron-BERT-345M-uncased models trained using the ‘out-of-fold’ method. In this approach, we combined the training and validation sets and then divided them randomly into five subsets. To train each model, we used four of these subsets as the training set and held out the fifth as a validation set. We used the checkpoint that performed best on the held-out set in each of the five runs in our final ensemble. At inference time, we aggregated the token class probabilities from each of the five models with equal weight and chose the token class with the highest probability. This ensemble achieved a strict F1-score of 0.764 on the unseen test set.

The second ensemble consisted of five different models: Megatron-BERT-345M-uncased, Megatron-BERT-345M-cased, BioMegatron-BERT-345M-uncased, RoBERTa-large and BERTweet-large. Each model was trained on the training set, and we used the checkpoint that performed best based on the validation set. To generate the final token labels, we calculated a weighted sum of the class probabilities produced by each model for each token. This ensemble achieved a strict F1-score of 0.753 on the unseen test set. The lower performance of this ensemble ran contrary to our expectation that an ensemble of different models trained on the same data would outperform an ensemble consisting of the same model type trained on different subsets of data. Our results suggest that performance on this task is limited more by the training data than by model architecture.

#### Team 4: Florida State University

Tweets consist of a large amount of nonstandard or semi-standard user-input sentences and characters including various special symbols and emojis. Therefore, we pre-processed all the tweets by (i) deleting all non-English characters, including emojis and special symbols that could not be decoded in American Standard Code for Information Interchange, and (ii) removing single characters if they were in the following list: ‘#$%&@+*\^‘/^~^’. To select our baseline model, we experimented and fine-tuned several commonly used pre-trained transformer models including BERT and its variants. We found that PubMedBERT (full text) achieved the highest F1 score, and used it as our baseline model. To cope with the highly unbalanced positive–negative case ratios, we used data augmentation to increase the proportion of positive cases and maximize the amount of information that could be extracted from limited labeled data. We considered three main data augmentation strategies: (i) augmenting true cases by replacing each original true entity with a randomly chosen medication mention from the set of medications mentioned in the training set; (ii) augmenting true cases by replacing each original true entity with a random string. We artificially created this string by randomly selecting 3–10 characters a–z and A–Z and (iii) augmenting true cases by randomly dropping a word that did not belong to a true entity in the tweet. Each of these strategies has its own advantages in terms of adding additional useful information for the model training.

We found that the combined augmentation strategy could help the model learn more about the context information and further improve its entity recognition capability. Our final system is a PubMedBERT-based classifier trained with a combination of multiple data augmentation approaches. Our method achieved satisfactory performance with an F1-score of 0.762 from the best submission, which compared favorably to the mean of all submissions with an F1-score of 0.696.

#### Team 6: Roche

Our approach (18) consisted of two steps: (i) develop models trained in different ways such that there is a higher diversity in their output and (ii) aggregate the outputs of the individual models using an ensemble approach to generate robust predictions. In our approach, we tried to address the problem of class imbalance with data augmentation and the problem caused by the unique characteristics of the language used on Twitter using three language models trained on tweets: Twitter Roberta (40) and two variants of BERTweet (28). We developed two different pipelines to fine-tune these models on the NER task using the BioCreative VII Track 3 challenge data. In both pipelines, we pre-trained the models on the SMM4H’18 dataset. The first pipeline used the challenge training data augmented by upsampling tweets that contained medication names. This pipeline performed hyper-parameter optimization to select the model that generates predictions on the test set. The second pipeline augmented the tweets that contain medication names by (i) tweet concatenation, (ii) tweet paraphrasing, (iii) medication name replacement and (iv) upsampling. In this pipeline, we created six different models trained on random subsets of the training data and then we aggregated the individual predictions by averaging.

We ran the first pipeline twice, each time with a different version of BERTweet (28) as a base model. We ran the second pipeline once using Twitter-RoBERTa (40) as a base model. We used an ensemble approach to make the final predictions on the test data based on the predictions from the three individual models by doing a character-level weighted sum of the model outputs and setting a predefined threshold to assign them to a medication name prediction. We treated model weights and the threshold as hyper-parameters, and we used both grid-search and optimization based on Tree of Parzen Estimators (41) to find the optimal values. Our objective function was the overlapping F1-score. Our ensemble approach achieved high performance on the overlapping evaluation (F1-score, 80.4%; precision, 82.7%; recall, 78.2%).

#### Team 7: DFKI, Technische Universität Berlin, Université Paris-Saclay

Our system relies on an ensemble model consisting of a transformer model and a data-driven approach using background knowledge, which uses known medication mentions from the given BioCreative training data, as well as the SMM4H dataset (11). Annotated medication mentions of those resources were extracted and then mapped to the (validation and) test set of the challenge. Using this approach, we are able to detect medication mentions reliably, but with a low recall.

In order to increase recall, we use a transformer model, more precisely, BioRedditBERT-uncased (BRB) (34). Originally, the model was initialized with BioBERT-Base (33) v1.0 + PubMed 200K + PubMed Central (PMC) 270K and then retrained with health-themed subreddits. Among different BERT implementations, BioRedditBERT achieved the best results in our preliminary experiments. The model was then fine-tuned on the training tweets of the challenge, using the Huggingface framework. Tweets were pre-processed with ekphrasis (42) for normalization (URL, email, time, numbers and dates) and tokenization. Moreover, an extra tokenization step was applied on the token level for words not included in the model’s vocabulary. The best results in the initial phase were achieved by labeling all resulting sub-tokens as a medication, instead of labeling the first subtoken only and disregarding the rest, as suggested in (32). The results of the transformer were then again post-processed in three successive stages. First, all sub-tokens resulting from the transformer tokenizer were merged with their corresponding labels into whole tokens again. Next, the spans of the predicted medications were identified, and finally, a custom span detection was applied, which merges all beginning medication tokens not separated by white spaces. The second step led to an increased strict F1-score.

The combination of both models (transformer+background knowledge) further improved the overall performance of our system. The best result of the combined model achieved a strict F1-score of 0.804 and an overlapping F1-score of 0.851 on the validation set. However, the model suffered a performance drop on the test set, which resulted in a strict F1-score of 0.725 (0.777 F1 overlap).

#### Team 8: Brigham Young University

After manually reviewing tweets mentioning medications and comparing them against multiple biomedical ontologies—including synonyms to terms in those ontologies—we found that most medications overlapped with existing ontology terms. In addition, we surmised that the lexicon of medications mentioned by pregnant women on social media would have a modest size, in part because pregnant women are advised against taking certain medications. We expected that misspellings and other variants would sometimes be captured by synonyms. Thus we decided to use a relatively simple, lexicon-based approach, which we expected would be relatively fast and interpretable.

We queried the training and validation sets and created a regular expression for each medication term identified, taking into account word boundaries and allowing for differences in casing. In addition, we removed hyperlinks and Twitter handles from the tweets. In our initial testing, we found that when medication terms were relatively short, they were often FPs, often because they were substrings of longer terms. Therefore, we sorted the terms from longest (highest number of characters) to shortest. After finding a match, we ignored tweets that partially matched a medication that had already been identified in a given tweet. Next, we augmented the lexicon with terms from the National Cancer Institute Thesaurus (43), focusing specifically on terms associated with the ‘Pharmacologic Substance’ category. Lastly, we used prior tweet examples to filter and augment our lexicon. We excluded terms that had been mentioned in tweets labeled as not being associated with medications more frequently than in tweets associated with medications. Furthermore, we added known positives from SMM4H’18 (11) to our lexicon; however, we did not exclude terms marked as negative in SMM4H because the biomedical domain was different. In post-validation testing, we attempted to augment our lexicon with terms from additional ontologies, but none of these attempts resulted in higher F1-scores.

Our simple approach performed moderately well in the validation phase of the competition, attaining a strict F1-score of 0.705 and an overlapping F1-score of 0.755. These scores were considerably lower than the top performers, which primarily used more sophisticated algorithmic approaches. However, our solution might be useful as an initial filter, identifying tweets that are most likely to mention medications and allowing more sophisticated algorithms to further discriminate between mentions and non-mentions. This approach could be useful as a way to reduce class imbalance to aid machine-learning approaches that are not suited for extremely imbalanced labels.

#### Team 9: University of Michigan

We built a DistilBERT model for this medication mention extraction task. DistilBERT is a model pre-trained with knowledge distillation, which retains 97% of the BERT performance but is 40% smaller and 60% faster (44). To encode the tokens, we used a pre-trained DistilBERT tokenizer on ready-split tokens rather than the full sentence. We also applied padding and truncation, to normalize the token sequence for each tweet to be the same length as the maximum sequence length in the dataset. DistilBERT uses WordPiece Tokenization, which can split single words into multiple tokens such that each token is likely to be in the vocabulary. To avoid the mismatch between labels and sub-tokens, we only train on the labels for the first sub-token of a split token.

We further evaluated whether bootstrapping would help improve DistilBERT models on the medication mention extraction task. To construct the training dataset for bootstrapping, we re-sampled the SMM4H’21 training data either five or ten times with replacement and combined the SMM4H’18 training data to each of them. We trained DistilBERT models on these re-sampled datasets and used them to predict the labels of the tweets in the validation and test sets. We used majority voting to ensemble the predictions of the models and determine the final predictions for each tweet.

The experiments show that the recall measure was consistently higher than precision in both validation and test sets. On the validation set, the bootstrapped model with the five-model ensemble achieved the highest overlapping F1 and strict F1-scores and outperformed the model with no bootstrapping. On the test set, we observed that while bootstrapped model still performed better than the individual model, the bootstrapped model with the ten-model ensemble achieved the best overlapping F1-score of 0.777 and the strict F1 score of 0.696. Through these experiments, we provided additional evidence to indicate that bootstrapped sampling helps further improve DistilBERT models for extracting medication mentions from health-related tweets.

#### Team 10: Georgia State University

We utilized a weak supervision approach (22) and trained several machine learning models with additional data beyond the provided training data. We utilized a silver standard dataset Tekumalla et al.(45), a gold standard dataset Klein et al.(46) and a medication dataset Sarker et al.(3) for data augmentation along with the Biocreative Training (BT) data. We used several training sizes by incrementally increasing the samples of medication tweets in the datasets and trained several machine learning models in a binary classification setting. We experimented with five classical models (Support Vector Machine, Naive Bayes, Decision Tree, Random Forest and Logistic Regression) and five deep learning models (Convolutional Neural Network, Long Short-Term Memory network, BERT, BioBERT and RoBERTa). To evaluate our models, we used the validation dataset and used the best models (BERT) trained on (BT + Klein *et al.* (46) + Sarker *et al.* (3)) and (BT + Tekumalla *et al.* (45) + Klein *et al.* (46) + Sarker *et al.* (3)) for our official submission since they obtained the best F-measure. We retrained the models adding the biocreative validation dataset and finally obtained the predictions on the test data. We filtered all the positive predictions and extracted the spans of the medication term using a medication dictionary (47). The SMMT_NER utility from the Social Media Mining Toolkit (48) was utilized for identifying the spans of the medication. Since the training data contain several terms that are not available in RxNorm (e.g. birth control), we computed a list of medication terms from the training and validation data and added it to our dictionary. We extracted the medication terms for all the tweets that were classified as medication tweets by the machine learning model. For a few of the tweets, we had to report the tweets as non-medication tweets although the model classified the tweet as a medication tweet due to the unavailability of the term in the dictionary. The BERT model trained with (BT + Klein *et al.* (46) + Sarker *et al.* (3)) datasets achieved the best results with an overlapping F1-score of 0.737 and strict F1 0.687 and was ranked 10 among all submissions.

#### Team 11: Concordia University

To address the task of medication name extraction, we compiled several gazetteer lists from the Medical Subject Headings (49) and DrugBank (50). Moreover, morphological information is encoded using a character-based CNN (51). The external information is leveraged together with ClinicalBERT embeddings (35) using the multi-input Recurrent Independent Mechanisms model (52), where each module is responsible for incorporating one information source. In addition to external resources, after the first training epoch, a Blacklist (FP terms) and a Whitelist (FN terms) are automatically generated by evaluating the model on the development set. Although the validation runs demonstrated consistent improvements for both precision and recall, the test results showed a low precision. Post-evaluation runs revealed that the greedily compiled Whitelist resulted in a poor precision, suggesting that such resources have to be further counterbalanced.

#### Team 12: University of Aveiro, University of A Coruña

We proposed an approach consisting of a deep learning solution based on transformer models to extract medication names from tweets. The core solution was further enhanced through the integration of simple pre- and post-processing mechanisms.

Regarding the model itself, the neural architecture of the solution consists of a language model (LM) followed by a multilayer perceptron (MLP) containing two fully connected layers (FCN) and a conditional random field (CRF) layer. For the language model, we opted for the publicly available RoBERTa models, which were already pre-trained on Twitter data (Available at https://huggingface.co/cardiffnlp/twitter-roberta-base). In the MLP, the first FCN uses the Mish (53) activation function and has 128 hidden units, whereas both the second FCN and the CRF layer have a size of *N*, where *N* corresponds to the number of possible tags. Here, *N* was set to 4 since the system used a modified version of the BIO tagging schema, where an additional tag named PAD was introduced in the schema to be used in padding tokens.

The language model was frozen during model training, therefore representing textual information using static contextualized word embeddings, and only the MLP and CRF layers were effectively trained. A weighted sample loss scheme was used to compensate for the imbalanced class distribution in the dataset, where negative samples (i.e. tweets where no ‘B’ or ‘I’ tag was detected) have their loss reduced by 60%, thus reducing their importance.

For the next step, a post-processing mechanism for reconstructing the predicted entities was introduced. More precisely, given that the RoBERTa model can split words into several sub-tokens, there may exist situations where the model wrongfully predicts only part of the ‘medication entity’ as an actual entity (e.g. tagging ‘xiety meds’ as an entity instead of ‘anti-anxiety meds’, which results in a negative match when using a strict evaluation). The method for token reconstruction assumes that when a model partially tags an entity, the remaining ill-classified entity sub-tokens should also be tagged as entity tokens. Unfortunately, after the official results were published, we noticed that the implementation of the token reconstructor had certain flaws related to how the RoBERTa model tokenized emojis into multiple sub-tokens, which negatively impacted the reconstructor and the final model performance.

During our challenge participation, we submitted three different runs, one of which did not use the reconstructor heuristic. Due to the previously mentioned error, the run without the reconstructing mechanism resulted in the team’s best approach, attaining a strict evaluation F1-score of 0.6810.

#### Team 13: Universidad Carlos III de Madrid

In this work, we experimented and fine-tuned several commonly used pre-trained deep neural models: BiLSTM+CRF and BERT. We adapted the NeuroNER model proposed in (54) for NER offset and entity classification of the BioCreative VII Track 3. Specifically, we have extended NeuroNER by adding contextualized-word information and information about overlapping or nested entities. Moreover, in this work, we used existing pre-trained noncontextualized-word models as well as our trained from scratch contextualized-word model: (i) a Glove 6B Embedding model (55), trained on Wikipedia and GoogleNews; (ii) word2vec PubMed-and-PMC-w2v trained on PubMed and PMC articles; (iii) the FastText English Twitter 100d trained on general tweets posts; (iv) our English medical word embeddings trained using the FastText model and (v) a sense-disambiguation embedding model (56). Finally, we fine-tune BERT pre-trained contextualized-word model and our trained from scratch contextualized-word model: (i) BioBERT-Large v1.1 trained on PubMed and PMC articles and (ii) BERTweet trained on general tweets and tweets mentioning the virus COVID-19. Our contribution consists of extending the NeuroNER system with additional features. In particular, contextualized-word representations and the BMEWO-V encoding format have been added to the network. BMEWO-V is similar to other previous encoding formats but allows the representation of nested and discontinuous entities. This format distinguishes the B tag for entity start, the M tag for entity continuity, the E tag for entity end, the W tag for a single entity and the O tag for other tokens that do not belong to any entity. The V tag allows us to represent nested entities. To extend the unbalanced training dataset, we obtained a collection of tweets containing medication mentions extracted using Konplik’s text analytics technology (supported by MeaningCloud [https://www.meaningcloud.com]) customized with dictionaries containing medication names such as the Unified Medical Language System that helps us to filter out irrelevant posts. We joined this collection to the CSIRO Adverse Drug Event Corpus (CADEC) dataset (57) and the TwiMed collection (4) to create a dataset of 160 000 tweets containing a mention of a medication. We used tweets to create our own embedding model.

Both NeuroNER and BERT models have been evaluated on the BioCreative VII Track 3 dataset obtaining an F-measure of 64.2% and 67.7%, respectively. Experiment results on BioCreative VII Track 3 showed that our features representation improved each separate representation, implying that LSTM-based compositions play different roles in capturing token-level features for NER tasks, thus improving their combination. Moreover, specific domain contextualized word vector representations outperform general domain word vector representations.

#### Team 14: Chunghwa Telecom Laboratories

Identifying medical entities such as diseases and medications mentioned in short, informal and noisy social media texts such as tweets is challenging. We participated in Track 3 of BioCreative VII with the goal to extract the mentions of medications or dietary supplements in tweets. We used different solutions based on BERT and BiLSTM to develop our system under highly unbalanced data distribution. Four systems were developed for the task, the original BERT fine-tuned on the official training set, BERT with data augmentation (BERT-DA), a BiLSTM and a BiLSTM with the focal loss. Due to time constraints for predicting the labels of the test set, we only submitted the predictions of BERT and BERT-DA for evaluation. The best-performing model that we submitted was the BERT-DA, which obtained an F1-score of 70.4%. From the evaluation results, we confirmed the effectiveness of the proposed data augmentation method, which can greatly improve the recall of the developed system.

#### Team 15: Georgia State University

In our system, we used an ensemble approach to classify tweets that may mention drug/medication names. This ensemble consisted of the combination of three pre-trained transformer models: BERT (32), CT-BERT (58) and BioBERT (33). We fine-tuned the models using the training dataset provided by the organizers (59). As part of the system, a NER BERT model was implemented to predict the spans of the medications possibly mentioned in the tweets filtered by our classifier. The NER was trained using a combination of datasets (45, 59), from which labels were available. For example, if a token contained a one-token medication name, it was labeled as ‘DRUG’, whereas medication names with multiple tokens were labeled as ‘B-DRUG’ and ‘I-DRUG’, in which the first one represents the beginning of a token, including a medication mention, whereas the second one indicates the rest of the tokens that belong to the same medication mention.

With respect to the output from our system, it is important to point out that if multiple mentions are found, the row of the given tweet was duplicated for each mention. Overall, the ensemble model (for the classification process) internally outperformed each individual fine-tuned model, obtaining an F1-score of 88.18%. Moreover, for the extraction process of medication mentions, despite the imbalanced dataset used to classify these tweets, our system obtained an overlapping precision above 0.81 and a strict precision above 0.72. In the shared task, our overlapping F1-score of 0.659 was below the median average of all the participants in this task.

## Discussion

In all the systems but one, the transformer-based networks dominate the competition. However, it remains unclear from the results which type of corpora is the best for pre-training the embeddings. The 10 best systems chose input embeddings trained on corpora of various genres and domains. Some systems were trained on general domain corpora (e.g. Wikipedia and books), others on PubMed abstracts and PMC full-text articles, or a large number of tweets.

The most efficient architecture seems to rely on a filter to remove tweets unlikely to mention medication names and only perform the extraction on the tweets filtered in. Whereas the first-ranked system follows the strategy of the baseline system by training a dedicated classifier and applying it upstream from the extractor, the second-ranked system proposed a multi-task where the classification and the extraction were performed by the same neural network.

The main challenge of Task 3 was to train machine learning systems on the same balance of positives to negatives as found in Twitter timelines, with the ultimate application of such systems being precisely in the context of timelines. In past #SMM4H shared tasks for classification of tweets mentioning medications (not for the extraction of their spans), we observed a drop of 6.4 points in the F1-score between the best classifier of the #SMM4H shared task in 2018 working on a balanced corpus (60), from 0.918 F1-score to the 0.854 F1-score of the best classifier of the #SMM4H shared task in 2020 working on an imbalanced corpus (61), despite the strategy proposed to address the high degree of class imbalance (a combination of keyword-based pre-filter and an ensemble of classifiers trained with out-of-fold). To address the class imbalance of the BioCreative VII Task 3 corpus, most participants opted for data-level preprocessing methods and/or ensemble learning (8), with only one system experimenting with a cost-sensitive learning method with a weighted loss function (24).

Data-level preprocessing methods modify the distribution of the examples in the training set to improve the learning process. This can be done either by removing negative tweets, extending the initial training set with additional positive tweets or choosing a hybrid approach. Six systems chose lexicon-based filters or dedicated classifiers to remove negative tweets for this task, in essence detecting tweets not related to medical topics. Given the few positive examples in the training set, the most common approach was to add positive tweets, thus providing examples of the linguistic patterns where medications are mentioned.

Oversampling by duplicating positive tweets of the initial training set was used by only two systems. Data augmentation was the most popular method with 11 systems out of 16 using it. Besides adding the examples of the SMM4HBalancedDrug corpus, participants looked for existing corpora where medication names were annotated or easy to retrieve automatically. For example, the participants added examples from corpora annotated with adverse medication events (62) or self-report of medication intakes (11) released for previous #SMM4H shared tasks. They also proposed various heuristics to create a silver-standard corpus. The two main heuristics were to collect a large number of tweets and apply either a lexicon or an extractor trained on a small training corpus to extract the medication names. These additional tweets contained FP annotations; nonetheless, they were beneficial when the participants added them to the initial training set to (re-)train their extractors.

An alternative to data augmentation was to generate artificial tweets by modifying existing positive tweets. This method was chosen by three teams, two of them ranked in the top four positions. The most intuitive way to generate new tweets was to substitute the medication names mentioned in existing tweets with other medication names. The new medication name can be selected from the same medication class. Other methods used were concatenating two tweets into one or duplicating a tweet and distorting the duplicate by removing random words or characters. External tools were also used to paraphrase or translate the tweets first to German and then use the tweet after translating it back to English.

## Conclusion

In this paper, an overview of the results of Task 3 of BioCreative VII was presented. The task focuses on the extraction of medication or supplement names in the timelines of 212 Twitter users. Given a tweet posted by a user, the task consists of identifying the spans of text of all medication names mentioned in the tweet. Besides the colloquial style of tweets, the corpus presents an additional challenge to natural language processing systems since it exhibits the natural distribution of tweets of interest in a timeline, with a very low percentage of tweets mentioning medications or supplements. Among the 16 systems proposed for the task, the most popular approaches to improve learning on the imbalanced corpus were assembling different extractors and preprocessing the data to modify the distribution of the training examples. One key to success for the top-ranked systems was to filter out tweets unlikely to contain medication names with a dedicated classifier and identify the spans of medications on the remaining tweets with an extractor trained on a dataset extended with both real and artificially generated tweets mentioning medications.

The advance in natural language processing models, thanks mainly to transformers and the clever use of heuristics to rebalance the distribution of the training data, improved the performance of extractors when applied to a corpus of tweets with a high-class imbalance. With 0.804 strict F1-score, the performance of the best systems of this challenge is getting very close to the performance achieved by recent named entity recognizers when extracting from Twitter common named entities such as persons, locations and organizations (63).
